# Supplementary figures and images for: Integrative network pharmacology and machine learning identify potential targets of indole-3-lactic acid in colorectal cancer
Source: PLoS One. 2026 Mar 9;21(3):e0344478. doi: 10.1371/journal.pone.0344478 (PMC12970938; doi:10.1371/journal.pone.0344478)

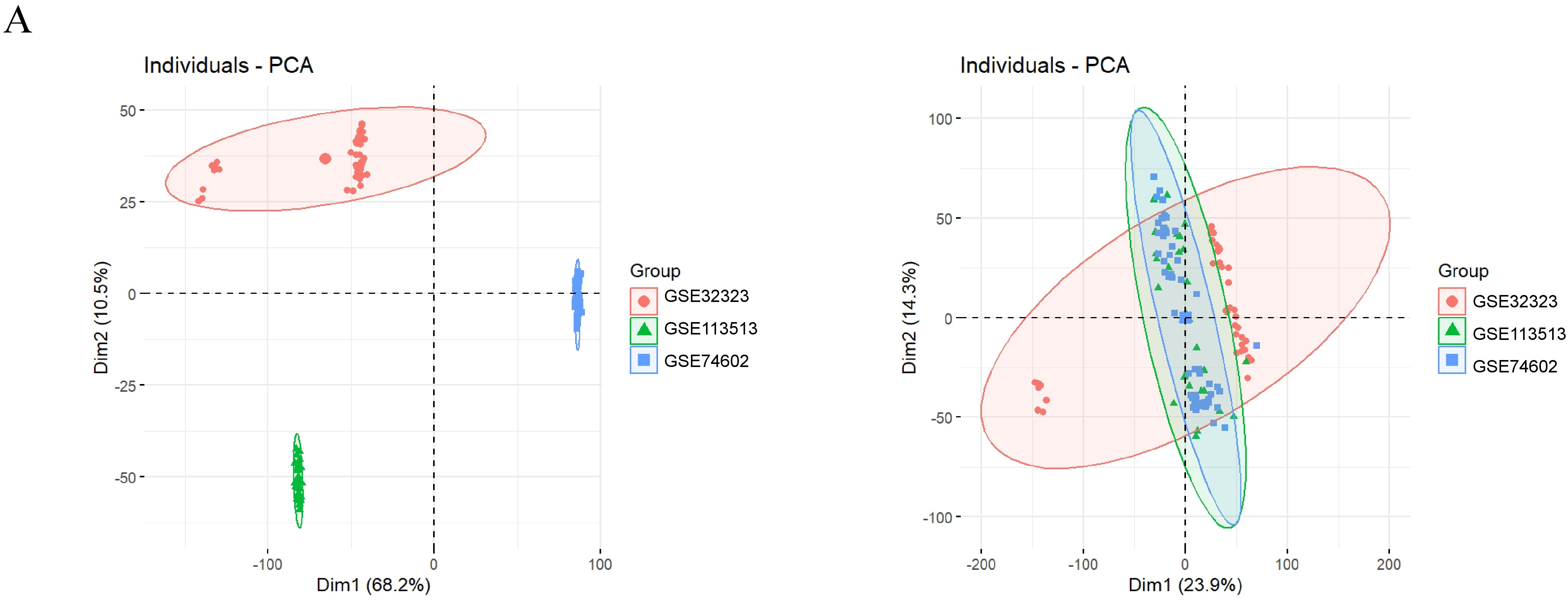

Supplement: S1 Data — S2 File. The summary of targets of CRC. S3 File. KEGG and GO analysis. S4 File. Feature importance rankings of hub genes. S1 Fig. The PCA plots before and after batch correction. S2 Fig. KEGG and GO analysis of 39 common targets. (ZIP) [file pone.0344478.s001.zip › Supporting Information/S1_Fig.tif]

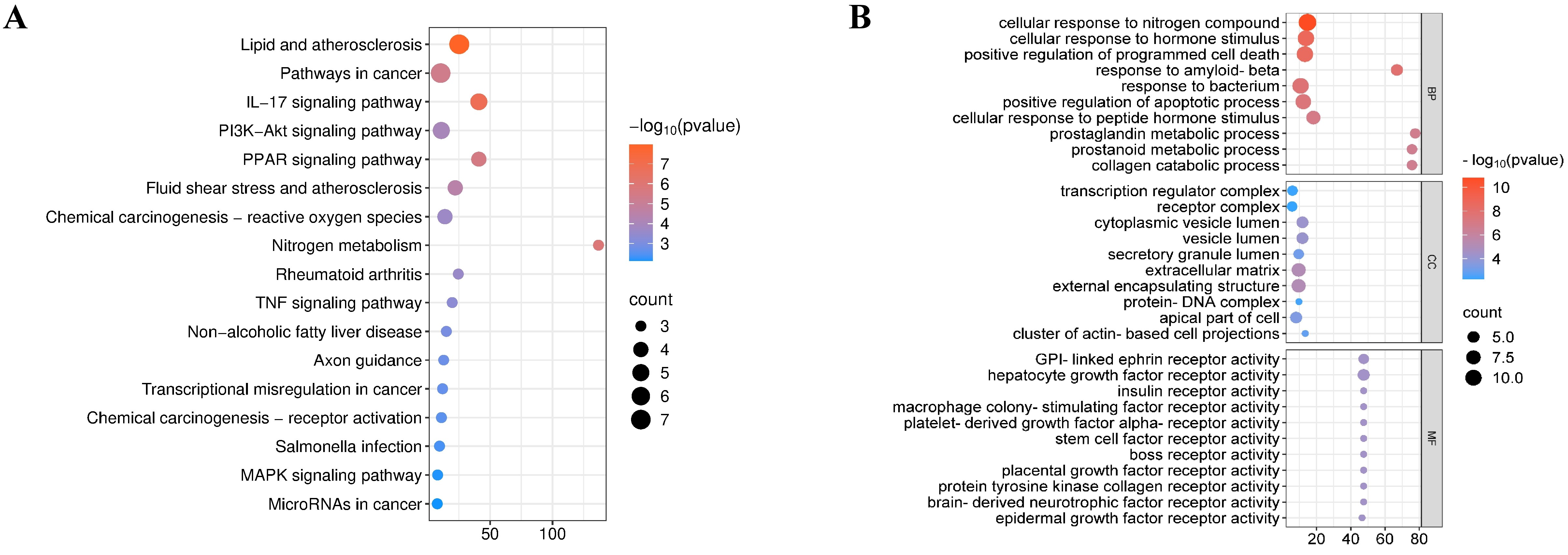

Supplement: S1 Data — S2 File. The summary of targets of CRC. S3 File. KEGG and GO analysis. S4 File. Feature importance rankings of hub genes. S1 Fig. The PCA plots before and after batch correction. S2 Fig. KEGG and GO analysis of 39 common targets. (ZIP) [file pone.0344478.s001.zip › Supporting Information/S2_Fig.tif]
