# Supplementary material for: Integrative network pharmacology and machine learning identify potential targets of indole-3-lactic acid in colorectal cancer
Source: PLoS One. 2026 Mar 9;21(3):e0344478. doi: 10.1371/journal.pone.0344478 (PMC12970938; doi:10.1371/journal.pone.0344478)
Supplement: Attachment — Submitted filename: Reviewers comment.docx [file pone.0344478.s002.docx]

**Reviewer 1**

From introduction to conclusion, the manuscript is well-structured and clear. A gut microbiota-derived metabolite, Indole-3-lactic acid (ILA), may treat colorectal cancer (CRC). The authors present a complete history. The study's goals, methods, and results are organized. A more extensive description of several portions would improve clarity before acceptance.

Title

• The title is written in a passive voice, which can make it sound less engaging and less clear. Consider rephrasing the title.

• The title is a bit long and should be rephrased to be more concise.

• Consider rephrasing the title to focus more on the research question or the main findings.

Abstract:

• Rephrase the sentence "Our study systematically elucidated..." to be more concise and impactful.

• Consider adding a brief summary of the study's main findings.

Introduction:

• Add more specific details about the current state of CRC research and how ILA fits into this landscape.

• Reorganize the paragraph discussing ILA's biological activities and mechanisms to improve flow.

• Add transitional phrases or sentences to connect the ideas between paragraphs.

• Rephrase the sentence "In this study, we integrated..." to be more concise and focused on the main objective.

Materials and Methods:

• Provide more detail on the databases and tools used, including their versions and specific parameters.

• Clarify the criteria for selecting the soft threshold, β.

• Provide more information on the PPI network analysis, including visualization parameters.

• Justify the use of specific machine learning algorithms.

• Provide more detail on the molecular docking analysis, including specific parameters used, such as the grid size and ligand flexibility.

• The section mentions that MD simulations were performed using Desmond in the Schrödinger Suite 2021-4. However, it would be helpful to provide more information on the specific protocol used, such as the simulation time, temperature, and pressure. I invite authors to clarify the MD simulation protocol.

The authors should revise the Methods section to include all necessary details regarding parameter selection, statistical thresholds, and multiple testing corrections to allow for full assessment and reproducibility.

Results

• Some figures and tables are not clearly described or referenced in the text. For example, Figure S1 is mentioned in the text, but its content is not clearly described. Clarify the presentation of results, including figures and tables.

• The KEGG and GO enrichment analyses are performed on the 39 therapeutic targets. However, it would be helpful to provide more context on why these specific targets were chosen and how they relate to the overall study.

• The machine learning algorithms identify four hub genes (EPHA2, HMOX1, MMP3, and PARP1). However, it would be helpful to discuss the implications of these findings and how they relate to the overall study. I recommend authors to discuss the implications of the machine learning results

• The molecular docking and MD simulations are performed on the four hub genes. Nevertheless, it would be supportive to provide more detail on the specific parameters used and the results obtained.

• The study has several limitations, including the reliance on bioinformatic analyses and the need for further functional validation.

• Clarify the implications of the study's findings and how they relate to the overall research question.

• The text employs various terms to denote the same concept, including "ILA-associated targets" and "ILA-CRC shared targets." Consistent terminology throughout the text would be beneficial.

• The text indicates the utilization of four GEO datasets; however, it lacks clarity regarding the rationale for selecting these particular datasets and the processing methods employed.

• The text states that WGCNA was employed to identify gene modules associated with CRC; however, it lacks clarity regarding the analysis methodology and the parameters utilized.

• The study is significantly dependent on bioinformatic analyses, which may introduce biases and limitations. It is essential to address these potential biases and limitations more explicitly.

• Phrases like "Collectively", "Notably", “In conclusion”, and "Furthermore" are used frequently throughout the manuscript, which can make it sound like it was written by an AI model.

**Reviewer 2**

**Potential mechanism prediction of indole-3-lactic acid against colorectal cancer based on network pharmacology, machine learning and molecular docking**

**Overall Assessment**

This manuscript presents a comprehensive in silico investigation of the potential mechanisms by which the gut microbiota–derived metabolite indole-3-lactic acid (ILA) may influence colorectal cancer (CRC). The authors integrate network pharmacology, transcriptomic analysis, machine learning–based feature selection, molecular docking, and molecular dynamics (MD) simulations. Given the increasing interest in gut microbiota–derived metabolites and their roles in cancer biology, this study provides a computational framework to identify potential molecular targets and pathways associated with ILA. Thus, it offers a useful hypothesis-generating resource for future experimental studies. However, several major issues related to interpretation, methodological transparency, and overstatement of conclusions should be addressed before the manuscript can be considered suitable for publication.

**Major Comments**

**1. Overinterpretation of Computational Findings**

Throughout the Abstract, Results, Discussion, and Conclusion, the manuscript frequently implies confirmed “therapeutic effects” of ILA against CRC e.g.

- *“Our study systematically elucidated the potential therapeutic effect of ILA on CRC…”* (Abstract)
- *“ILA may exert its anti-CRC effects by targeting these proteins”* (Results 3.8)

Given that the study is entirely computational, these statements overstate the strength of the evidence.

**Recommendation:**
The authors should revise the language throughout the manuscript to clearly frame the findings as *predictive*, *hypothesis-generating*, or *putative mechanisms*. Replace “therapeutic effect” with **“predicted mechanism”**, **“putative targets”**, or **“hypothesized regulatory roles”**. Claims of therapeutic efficacy should be avoided unless supported by experimental validation.

**2. Target Prediction Strategy Requires Additional Justification**

In Section 2.1 (Collection of ILA targets), predicted targets from multiple databases are combined without reporting confidence scores, prediction probabilities, or overlap frequency across databases.

**Recommendation:**
The authors should provide additional information on target confidence, such as:

- The number of databases supporting each target
- Probability or score thresholds (where available)
- A rationale for including low-confidence predictions

**3. Machine Learning Methodology Lacks Transparency**

In Section 2.7, the use of LASSO, Random Forest, and SVM-RFE is appropriate; however, important methodological details are missing:

- Handling of class imbalance between normal and CRC samples is not described.
- Feature scaling or normalization prior to SVM analysis is not reported.
- The Random Forest importance threshold (>0.3) is not justified.

**Recommendation:**
The authors should clarify preprocessing steps, provide justification for parameter choices, and include additional information to ensure reproducibility. Reporting cross-validation strategies and performance metrics in greater detail would strengthen this section.

**4. Interpretation of Immune Infiltration Analysis**

The immune infiltration analysis (Section 3.7) identifies correlations between hub gene expression and immune cell proportions. However, the discussion occasionally implies mechanistic regulation of immune cell behavior by ILA or hub genes.

**Recommendation:**
The authors should explicitly state that these findings are correlational. Causal language should be avoided, and conclusions regarding immune modulation should be clearly framed as speculative since these claims was not substantially validated in an invitro or in vivo model.

**5. Molecular Docking and Molecular Dynamics Analysis**

The docking and MD simulations support binding plausibility between ILA and the identified hub proteins. However, the rationale for extending the MD simulation to 100 ns for only the ILA–HMOX1 complex, while limiting others to 10 ns, is not sufficiently explained.

**Recommendation:**
The authors should justify the selection of HMOX1 for extended simulation and discuss the limitations of short MD trajectories for the other complexes. Additional stability metrics or binding free energy calculations would further strengthen this section. Report RMSF, hydrogen bond occupancy, and binding free energy (MM-PBSA). Also Include control ligands or known inhibitors of CRC for benchmarking.

**Minor Comments**

1. **Data Availability Statement**
   The statement “All data are available on request from the authors” does not align with PLOS ONE data-sharing recommendations. Public access to analysis scripts (e.g., GitHub, Zenodo) or processed data should be provided where possible.
2. **Redundancy in the Discussion**
   Several mechanistic explanations (e.g., AhR/Nrf2 signaling) are repeated across multiple subsections. The Discussion could be streamlined to improve clarity.
3. **Language and Formatting**
   Minor grammatical errors and overly long sentences are present, particularly in the Discussion. Gene and protein nomenclature should be consistently formatted.
