# Supplementary material for: Integrative network pharmacology and machine learning identify potential targets of indole-3-lactic acid in colorectal cancer
Source: PLoS One. 2026 Mar 9;21(3):e0344478. doi: 10.1371/journal.pone.0344478 (PMC12970938; doi:10.1371/journal.pone.0344478)
Supplement: Attachment — Submitted filename: Response to Reviewers.doc [file pone.0344478.s004.doc]

Response to Reviewers and Editors

January 19, 2026

PONE-D-25-56178

Dear Editors:

Thank you for giving us the opportunity to submit a revised draft of our manuscript “Potential mechanism prediction of indole-3-lactic acid against colorectal cancer based on network pharmacology, machine learning and molecular docking” (Submission ID PONE-D-25-56178) for publication in PLOS One. We appreciate the time and effort that you and the reviewers dedicated to providing feedback on our manuscript and are grateful for the insightful comments and valuable improvements to our paper. We have read your comments carefully and have made some modifications which hopefully could meet your expectations. **The revised portions are highlighted in red in the manuscript.** The main modifications in the paper are summarized and the response to the reviewers’ comments are as follows:

**Reviewer 1, Point 1**

1. Comment: (Title: The title is written in a passive voice, which can make it sound less engaging and less clear. Consider rephrasing the title. The title is a bit long and should be rephrased to be more concise. Consider rephrasing the title to focus more on the research question or the main findings.)

**Response:** We sincerely appreciate your thorough review and valuable suggestions regarding our manuscript. Following your advice, we have rephrased the title to be more concise, active, and focused on the core research findings. We have revised the original title to: “Integrative network pharmacology and machine learning identify potential targets of indole-3-lactic acid in colorectal cancer”. We have updated the title on the Title Page and throughout the manuscript submission system.

**Reviewer 1, Point 2**

2. Comment: (Abstract: Rephrase the sentence "Our study systematically elucidated..." to be more concise and impactful. Consider adding a brief summary of the study's main findings.)

**Response:** We sincerely thank your for this insightful suggestion regarding the Abstract. Following your suggestion, we have carefully revised the Abstract and rewritten the concluding sentence to be more concise and impactful. The revised sentence reads as follows: “In conclusion, these results highlight EPHA2, HMOX1, MMP3, and PARP1 as candidate targets and suggest that ILA may influence CRC-related signaling, metabolic programs, and immune contexture, providing a theoretical foundation for developing gut microbiota-derived metabolites as novel anticancer strategies”. We have updated the Abstract in the revised manuscript.

**Reviewer 1, Point 3**

3. Comment: (Introduction: Add more specific details about the current state of CRC research and how ILA fits into this landscape.)

**Response:** We sincerely thank your for this insightful suggestion regarding the Introduction. Following your suggestion, we have rewritten the Introduction, with the main revisions focusing on adding and strengthening the following two aspects: **(1)** Summarize the current therapeutic and research challenges in CRC, including molecular heterogeneity and treatment resistance. **(2)** Better position gut microbiota–derived metabolites as an emerging research direction relevant to tumor metabolism and the immune microenvironment. We further clarified how ILA, as a tryptophan-derived microbial metabolite with reported anti-inflammatory, antioxidant and immunomodulatory activities, conceptually fits this framework and why a systematic target- and pathway-level investigation is needed. These revisions have been incorporated in the **Introduction section**. Please see the specific modifications in the revised manuscript.

**Reviewer 1, Point 4**

4. Comment: (Materials and Methods: Provide more detail on the databases and tools used, including their versions and specific parameters.)

**Response:** Thank you very much for pointing this out, and we think this is an excellent suggestion. In accordance with your suggestion, we have thoroughly updated the "Materials and Methods" section to ensure full transparency and reproducibility. The specific revisions are as follows: **(1)** Database access: We have added the specific URLs and last access dates for all online databases. **(2)** Software versions: Beyond the main R software version (v4.4.2), we have now specified the exact version numbers for all critical R packages used in the analysis. **(3)** Parameter specification: We have elaborated on the specific parameters for the computational models used in this study. Specifically, we provided the detailed workflow for the PPI network analysis (**Section 2.5**); clarified the rationale for the selected machine learning algorithms alongside their hyperparameter tuning settings (**Section 2.7**); and supplemented detailed information regarding the molecular docking and molecular dynamics simulations (**Sections 2.10 and 2.11**). These modifications have significantly improved the clarity and readability of our manuscript. Please see the specific modifications in the revised manuscript.

**Reviewer 1, Point 5**

5. Comment: (Materials and Methods: Clarify the criteria for selecting the soft threshold, β.)

**Response:** Thank you for your pointing this out. We apologize for not making it clear. We have added a detailed description in **Section 2.4** of the criteria used to select the soft-thresholding power (β) in the WGCNA analysis, as follows: “We determined the optimal soft threshold power (β) using the pickSoftThreshold function in the WGCNA R package. The criterion for selection was to achieve a scale-free topology fit index (R2) of at least 0.85 while maintaining a reasonable mean connectivity. Consequently, a power of β=X was selected based on the scale independence and mean connectivity plots. ”

**Reviewer 1, Point 6**

6. Comment: (Materials and Methods: Provide more information on the PPI network analysis, including visualization parameters.)

**Response:** Thank you for your pointing this out. We apologize for not making it clear. We have added a detailed description in **Section 2.5** of the parameters and visualization workflow used for the PPI analysis, as follows: “The PPI data were downloaded from the STRING database in TSV format and imported into Cytoscape (version 3.9.1) for network construction and visualization. Network topological properties were analyzed using the ‘Analyze Network’ tool with the network treated as undirected (‘Treat network as undirected’). Node centrality measures were then calculated using the CytoNCA plugin (version 2.1.6), with degree centrality used as the primary metric. Isolated nodes without interactions (degree = 0) were excluded from the network. Finally, the ‘Style’ panel was used to map node attributes for visualization: node size and color were scaled according to degree centrality values, with larger and darker nodes indicating higher connectivity.”

**Reviewer 1, Point 7**

7. Comment: (Materials and Methods: Justify the use of specific machine learning algorithms.)

**Response:** Thank you very much for your comments. We have added a detailed justification in **Section 2.7** for using the three machine-learning algorithms (LASSO, RF, and SVM-RFE), as follows: “Specifically, LASSO employs L1 regularization to mitigate multicollinearity and induce sparsity in high-dimensional datasets; RF was selected to capture complex non-linear interactions; and SVM-RFE identifies an optimal feature subset to maximize classification performance. These three algorithms are complementary, and their combined use reduces the bias inherent to any single model, thereby improving the robustness of the screening results.”

**Reviewer 1, Point 8**

8. Comment: (Materials and Methods: Provide more detail on the molecular docking analysis, including specific parameters used, such as the grid size and ligand flexibility.)

**Response:** We sincerely appreciate the valuable comments. In response to your suggestion, we have updated **Section 2.10** with the following detailed parameters: **(1)** Grid generation: The receptor grids were generated using the Receptor Grid Generation panel in Glide. The center of the grid box was defined by the centroid of the co-crystallized ligand within the active site of each target protein. The bounding box was sized to sufficiently enclose the active site and accommodate the ligand. The specific grid dimensions for each target are provided in **Table 2**. **(2)** Ligand flexibility: The Glide Standard Precision mode was employed, which internally generates multiple conformations for the ligand. The sampling included the exploration of ring conformations and nitrogen inversions. To account for protein flexibility implicitly and allow for minor steric clashes, the van der Waals radii of the ligand atoms were scaled by a factor of 0.80 with a partial charge cutoff of 0.15, while the receptor atoms were scaled by 1.0. **(3)** The resulting poses were subjected to post-docking minimization, and the best-scored pose (lowest GlideScore) was selected for further analysis. These revisions have been incorporated into the manuscript **Section 2.10**.

**Reviewer 1, Point 9**

9. Comment: (Materials and Methods: The section mentions that MD simulations were performed using Desmond in the Schrödinger Suite 2021-4. However, it would be helpful to provide more information on the specific protocol used, such as the simulation time, temperature, and pressure. I invite authors to clarify the MD simulation protocol.)

**Response:** Thank you very much for pointing this out, and we agree that a clearer and more detailed description of the MD protocol is essential for reproducibility. We have therefore revised **Section 2.11** to explicitly report the key simulation settings, including the simulation time for each system, the ensemble, temperature, and pressure, as well as additional protocol details (system solvation, equilibration procedure, time step and constraints, thermostat, and trajectory recording frequency). Briefly, all simulations were performed in Desmond (Schrödinger Suite 2021-4) using the OPLS4 force field, in a TIP3P water box with 0.15 M NaCl. After minimization and equilibration, four independent 10 ns comparative simulations were conducted for the four protein-ligand complexes, and a 100 ns production run was performed for the ILA-HMOX1 complex under the NPT ensemble at 300 K and 1 atm. These details have been added to the revised manuscript **Section 2.11**.

**Reviewer 1, Point 10**

10. Comment: (Materials and Methods: The authors should revise the Methods section to include all necessary details regarding parameter selection, statistical thresholds, and multiple testing corrections to allow for full assessment and reproducibility.)

**Response:** We sincerely appreciate the valuable comments. Accordingly, we have comprehensively revised the Materials and Methods section to explicitly describe the criteria and parameter settings used at each analytical step. We have incorporated these revisions into **Section 2** of the revised manuscript.

**Reviewer 1, Point 11**

11. Comment: (Results: Some figures and tables are not clearly described or referenced in the text. For example, Figure S1 is mentioned in the text, but its content is not clearly described. Clarify the presentation of results, including figures and tables.)

**Response:** Thank you for your pointing this out. We apologize for not making it clear. In accordance with your suggestion, we have carefully re-examined the entire **Results section**. **(1)** Regarding Figure S1: We have added a detailed description in the main text to explain the changes in PCA plots before and after batch effect correction, demonstrating the reliability of our data integration. **(2)** General revision: We have systematically checked the citations and descriptions for all figures (Figures 1-11; Figure S1-S2) and tables (Tables 1-2). We revised the manuscript to ensure that every reference to a figure or table is accompanied by a clear interpretation of the specific data trends and their biological significance. Please see the specific modifications in the revised manuscript.

**Reviewer 1, Point 12**

12. Comment: (The KEGG and GO enrichment analyses are performed on the 39 therapeutic targets. However, it would be helpful to provide more context on why these specific targets were chosen and how they relate to the overall study.)

**Response:** We sincerely appreciate the valuable comments.

**(1)** The 39 targets used for GO and KEGG enrichment analyses were not arbitrarily selected but were identified through a stepwise integrative strategy designed to link ILA with CRC in a biologically meaningful manner. Specifically, these 39 targets represent the intersection between 1) putative ILA-related targets predicted using multiple network pharmacology databases and 2) CRC key genes defined by integrating disease-related databases, differential expression analysis, and WGCNA. Therefore, these targets simultaneously reflect the potential molecular actions of ILA and the core pathological features of CRC.

**(2)** Performing functional enrichment analyses on this refined target set allowed us to focus on biological processes and signaling pathways that are most likely involved in the therapeutic effects of ILA against CRC, rather than generating nonspecific results from broader gene sets. Importantly, the enriched GO terms and KEGG pathways provide mechanistic context for the subsequent identification of hub genes, immune infiltration analysis, and molecular docking.

**(3)** To clarify this rationale, we have revised the Results section (**Section 3.3**) and added explanatory text describing the selection criteria and biological significance of the 39 therapeutic targets, as well as their role in the overall study framework.

**Reviewer 1, Point 13**

13. Comment: (The machine learning algorithms identify four hub genes (EPHA2, HMOX1, MMP3, and PARP1). However, it would be helpful to discuss the implications of these findings and how they relate to the overall study. I recommend authors to discuss the implications of the machine learning results.)

**Response:** Thank you very much for your comments. In this study, machine learning algorithms were applied not merely as a feature-selection tool but as an integrative strategy to identify the most robust and biologically relevant hub genes among the potential therapeutic targets of ILA against CRC. By combining LASSO regression, RF, and SVM-RFE, we aimed to reduce model-specific bias and improve the reliability of hub gene selection.

**(1)** The four hub genes identified were consistently supported by multiple independent analyses, including PPI network topology, differential expression and diagnostic performance, gene set enrichment analysis, immune infiltration correlations, and molecular docking and molecular dynamics simulations. This convergence of evidence indicates that these genes represent key molecular nodes linking ILA-associated targets to CRC-related pathological processes, rather than being isolated machine learning outputs.

**(2)** From a biological perspective, these hub genes are involved in critical processes relevant to CRC progression. Their identification therefore provides mechanistic insight into how ILA may exert anti-CRC effects through coordinated regulation of inflammation, oxidative stress, metabolism, and tumor-host interactions.

**(3)** We have added a comprehensive discussion at the end of **Section 4.1** of the Discussion to improve the interpretability and biological relevance of the machine learning analyses.

**Reviewer 1, Point 14**

14. Comment: (The molecular docking and MD simulations are performed on the four hub genes. Nevertheless, it would be supportive to provide more detail on the specific parameters used and the results obtained.)

**Response:** We sincerely appreciate the valuable comments. In accordance with your recommendation, we have made substantial additions to the manuscript in two areas: **(1)** We have supplemented the text with specific operational parameters for molecular docking and molecular dynamics simulations in the Methods section (**Sections 2.10 and 2.11**). **(2)** We have provided a more granular and quantitative description of the simulation outcomes in the Results section (**Sections 3.8 and 3.9**). We believe these added details significantly enhance the transparency and robustness of our computational findings.

**Reviewer 1, Point 15**

15. Comment: (The study has several limitations, including the reliance on bioinformatic analyses and the need for further functional validation.)

**Response:** We sincerely appreciate your thorough review and valuable suggestions regarding our manuscript. We fully acknowledge that the present study is primarily based on bioinformatic, network pharmacology, and machine learning analyses, and that further experimental validation is required to confirm the functional roles of the identified targets and pathways. **(1)** Although our study relies on computational approaches, we employed a multi-layered integrative strategy to enhance the robustness and reliability of the findings. This included the integration of multiple target prediction databases, transcriptomic analyses, complementary machine learning algorithms, external dataset validation, immune infiltration analysis, as well as molecular docking and molecular dynamics simulations. Together, these approaches provide converging evidence that supports the biological relevance of the identified hub genes and pathways. Its primary contribution lies in providing a high-confidence theoretical foundation for subsequent experimental studies, thereby narrowing validation scope and improving research efficiency. **(2)** Future studies will focus on in vitro and in vivo experiments to validate the regulatory effects of ILA on the identified hub genes and signaling pathways, and to further elucidate their roles in CRC progression. **(3)** We have revised the Discussion section (**Section 4.4**) to explicitly acknowledge these limitations and to outline directions for future experimental validation.

**Reviewer 1, Point 16**

16. Comment: (Clarify the implications of the study's findings and how they relate to the overall research question.)

**Response:** We sincerely appreciate the valuable comments. The overall research question of this study was to systematically explore the potential molecular mechanisms by which the gut microbiota-derived metabolite ILA may exert therapeutic effects in CRC. Our findings address this question at multiple levels. **(1)** First, by integrating network pharmacology with transcriptomic analyses, we identified a refined set of potential therapeutic targets linking ILA to CRC-related molecular alterations. **(2)** Second, machine learning approaches further prioritized four robust hub genes that represent key molecular nodes within this network. **(3)** Third, functional enrichment and gene set enrichment analyses revealed that these targets are involved in biological processes and signaling pathways closely associated with CRC progression, including inflammation, oxidative stress, metabolism, and tumor microenvironment regulation. **(4)** Finally, molecular docking and molecular dynamics simulations provided structural support for potential interactions between ILA and the identified hub proteins. Collectively, the integrative framework presented here highlights the potential of gut microbiota-derived metabolites as sources of adjuvant therapeutic strategies for CRC. We have revised the **Conclusion section** to more explicitly connect the main findings with the overall research question and to clarify the broader implications of the study.

**Reviewer 1, Point 17**

17. Comment: (The text employs various terms to denote the same concept, including "ILA-associated targets" and "ILA-CRC shared targets." Consistent terminology throughout the text would be beneficial.)

**Response:** Thank you for your pointing this out. We apologize for not making it clear. To address this issue, we have conducted a thorough review of the entire text and rigorously standardized the nomenclature for the distinct gene sets involved in our study. We have defined and unified the terms as follows: **(1)** ILA-related targets: Refers specifically to the 626 ILA-associated targets retrieved from public databases.

CRC-related targets: Refers specifically to the colorectal cancer-associated genes identified through database mining and WGCNA analysis. **(2)** ILA-CRC common targets: Refers specifically to the intersection of ILA and CRC genes, comprising 39 genes. **(3)** Hub genes: Refers specifically to the four hub genes (EPHA2, HMOX1, MMP3, and PARP1) ultimately identified. Please see the specific modifications in the revised manuscript.

**Reviewer 1, Point 18**

18. Comment: (The text indicates the utilization of four GEO datasets; however, it lacks clarity regarding the rationale for selecting these particular datasets and the processing methods employed.)

**Response:** We sincerely appreciate the valuable comments. **(1)** Rationale for dataset selection: We conducted a comprehensive search in the GEO database with the following strict inclusion criteria: 1) Organism restricted to Homo sapiens; 2) Data type restricted to gene expression microarrays; 3) The dataset must contain both primary colorectal cancer tissues and normal colonic tissues; 4) A minimum sample size (>25) to ensure statistical power. Based on these criteria, GSE44076 was designated as the training set due to its relatively large sample size (98 tumor, 50 normal) and high data quality, making it ideal for constructing the WGCNA network and identifying differentially expressed genes (DEGs). The other three datasets (GSE74602, GSE32323, and GSE113513) were selected as external validation sets to test the diagnostic performance of the hub genes across different platforms and populations, thereby ensuring the generalizability of our findings. The details of the dataset are shown in **Table 1**. **(2)** Data processing methods: Raw expression data were obtained from the GEO database and subjected to standard preprocessing procedures, including background correction and normalization. Differential expression analysis was performed using established bioinformatic pipelines with consistent statistical thresholds across all datasets. We have rewritten **Section 2.2** in the manuscript to explicitly list the inclusion criteria and detail the batch effect correction process.

**Reviewer 1, Point 19**

19. Comment: (The text states that WGCNA was employed to identify gene modules associated with CRC; however, it lacks clarity regarding the analysis methodology and the parameters utilized.)

**Response:** Thank you for your pointing this out. We apologize for not making it clear. In this study, WGCNA was performed using the WGCNA package in R to identify gene modules associated with CRC. We have rewritten **Section 2.4** to explicitly include these parameters and clarify the workflow.

**Reviewer 1, Point 20**

20. Comment: (The study is significantly dependent on bioinformatic analyses, which may introduce biases and limitations. It is essential to address these potential biases and limitations more explicitly.)

**Response:** We sincerely appreciate the valuable comments. We fully acknowledge that the present study is primarily based on bioinformatic, network pharmacology, and machine learning analyses, and that further experimental validation is required to confirm the functional roles of the identified targets and pathways. **(1)** Although our study relies on computational approaches, we employed a multi-layered integrative strategy to enhance the robustness and reliability of the findings. This included the integration of multiple target prediction databases, transcriptomic analyses, complementary machine learning algorithms, external dataset validation, immune infiltration analysis, as well as molecular docking and molecular dynamics simulations. Together, these approaches provide converging evidence that supports the biological relevance of the identified hub genes and pathways. Its primary contribution lies in providing a high-confidence theoretical foundation for subsequent experimental studies, thereby narrowing validation scope and improving research efficiency. **(2)** Future studies will focus on in vitro and in vivo experiments to validate the regulatory effects of ILA on the identified hub genes and signaling pathways, and to further elucidate their roles in CRC progression. **(3)** We have revised the Discussion section (**Section 4.4**) to explicitly acknowledge these limitations and to outline directions for future experimental validation.

**Reviewer 1, Point 21**

21. Comment: (Phrases like "Collectively", "Notably", “In conclusion”, and "Furthermore" are used frequently throughout the manuscript, which can make it sound like it was written by an AI model.)

**Response:** We sincerely appreciate the thoughtful comments. To address this, we have conducted a thorough manual proofreading and revision of the entire text, focusing on improving sentence variety and linguistic flow. Specifically, we minimized the repeated use of these expressions by restructuring sentences, varying syntactic constructions, and replacing them with more neutral or context-appropriate transitions where necessary. We believe that the revised version now reads more naturally and aligns better with standard academic writing conventions.

**Reviewer 2, Point 1**

1. Comment: (The authors should revise the language throughout the manuscript to clearly frame the findings as predictive, hypothesis-generating, or putative mechanisms. Replace “therapeutic effect” with “predicted mechanism”, “putative targets”, or “hypothesized regulatory roles”. Claims of therapeutic efficacy should be avoided unless supported by experimental validation. )

**Response:** We sincerely appreciate this important and constructive comment. We fully agree that, given the computational and bioinformatic nature of this study, the findings should be framed as predictive and hypothesis-generating rather than as direct evidence of therapeutic efficacy. In response to this suggestion, we have carefully revised the language throughout the manuscript to adopt more cautious and precise terminology. In addition, we have emphasized in the **Discussion and Limitations sections** that the proposed mechanisms and targets are based on integrative bioinformatic analyses and require further experimental validation. We believe that these changes improve the scientific rigor and clarity of the manuscript while avoiding overinterpretation of the results. Please refer to the revised manuscript for all modifications.

**Reviewer 2, Point 2**

2. Comment: (In Section 2.1 (Collection of ILA targets), predicted targets from multiple databases are combined without reporting confidence scores, prediction probabilities, or overlap frequency across databases. The authors should provide additional information on target confidence, such as: The number of databases supporting each target; Probability or score thresholds (where available); A rationale for including low-confidence predictions)

**Response:** We sincerely appreciate the valuable comments. To enhance data transparency and address your concerns, we have implemented the following revisions: **(1)** Clarification of thresholds: We have updated **Section 2.1** of the manuscript to explicitly state the screening parameters used for each database. **(2)** Reporting overlap frequency: to provide transparency regarding prediction confidence, detailed information on the source databases and the overlap frequency (the number of databases supporting each target) for all 626 unique targets is listed in **Supplementary File 1**. **(3)** Funnel screening strategy: our study design employs a funnel-like approach. The initial step aims to maximize sensitivity to capture the full pharmacological landscape and avoid missing novel targets. The subsequent steps-intersection with rigorously defined CRC targets, followed by machine learning consensus and molecular docking. Therefore, the inclusive approach at the data collection stage ensures the comprehensiveness of the final hub gene identification. Please refer to the revised manuscript for all modifications.

**Reviewer 2, Point 3**

3. Comment: (In Section 2.7, the use of LASSO, Random Forest, and SVM-RFE is appropriate; however, important methodological details are missing: Handling of class imbalance between normal and CRC samples is not described; Feature scaling or normalization prior to SVM analysis is not reported. The Random Forest importance threshold (>0.3) is not justified. The authors should clarify preprocessing steps, provide justification for parameter choices, and include additional information to ensure reproducibility. Reporting cross-validation strategies and performance metrics in greater detail would strengthen this section.)

**Response:** Thank you very much for your comments. We fully agree that providing detailed descriptions of data preprocessing, justifications for parameter choices, and model evaluation strategies is essential for ensuring the robustness and reproducibility of our machine learning analysis. Following your suggestions, we have comprehensively updated the methodology section: **(1)** Class imbalance: We have clarified the sample composition in the training dataset. we used a stratified cross-validation scheme to preserve the normal/CRC ratio within each fold. **(2)** Feature scaling prior to SVM-RFE: We have added a detailed description of preprocessing for SVM-RFE.we performed Z-score standardization (zero mean and unit variance) on the gene expression matrix prior to the SVM-RFE analysis. **(3)** Justification for RF Threshold: The importance threshold (>0.3) was selected based on the distribution of the feature importance scores (Supplementary File 4). This value served as an empirical cutoff to retain the top-tier genes that contributed most significantly to the classification while filtering out low-contribution noise. **(4)** We have expanded **Section 2.7** to report the cross-validation procedures and key parameter settings for LASSO, Random Forest, and SVM-RFE (including the number of folds, repetition where applicable, and random seed settings). Please refer to the revised manuscript for all modifications.

**Reviewer 2, Point 4**

4. Comment: (The immune infiltration analysis (Section 3.7) identifies correlations between hub gene expression and immune cell proportions. However, the discussion occasionally implies mechanistic regulation of immune cell behavior by ILA or hub genes. The authors should explicitly state that these findings are correlational. Causal language should be avoided, and conclusions regarding immune modulation should be clearly framed as speculative since these claims was not substantially validated in an invitro or in vivo model.)

**Response:** We sincerely appreciate the valuable comments. To ensure the scientific rigor of our conclusions, we have carefully re-examined **Section 3.7** (Results) and **Section 4.3** (Discussion) and implemented the following revisions: **(1)** We have replaced terms implying "mechanistic regulation" ("regulate," "drive," "determine") with terms describing "statistical association" ("correlate with," "is associated with," "suggest a potential link"). **(2)** In the discussion regarding the immune microenvironment, we have explicitly stated that these findings are speculative inferences based on bioinformatic predictions. We have emphasized that these potential interactions require confirmation through future in vitro and in vivo experiments. Please see the specific modifications in the revised manuscript.

**Reviewer 2, Point 5**

5. Comment: (The docking and MD simulations support binding plausibility between ILA and the identified hub proteins. However, the rationale for extending the MD simulation to 100 ns for only the ILA-HMOX1 complex, while limiting others to 10 ns, is not sufficiently explained. The authors should justify the selection of HMOX1 for extended simulation and discuss the limitations of short MD trajectories for the other complexes. Additional stability metrics or binding free energy calculations would further strengthen this section. Report RMSF, hydrogen bond occupancy, and binding free energy (MM-PBSA). Also Include control ligands or known inhibitors of CRC for benchmarking.)

**Response:** We sincerely appreciate the constructive and technically insightful comment. To enhance data transparency and address your concerns, we have implemented the following revisions:

**(1)** Rationale for simulation duration: we first performed 10 ns simulations for all four hub gene complexes to assess initial structural stability. The results indicated that the ILA-HMOX1 pose exhibited favorable interaction geometry and stable intermolecular contacts in preliminary short simulations. Based on the stability profile from the screening phase and the biological context (HMOX1 is the central effector in the oxidative stress and ferroptosis pathways mediated by ILA, aligning with our enrichment analysis), we selected HMOX1 for an extended 100 ns simulation to capture long-timescale dynamics. We have added this justification in the revised manuscript (**Section 3.9**).

**(2)** Limitations of short MD trajectories: We agree that 10 ns trajectories mainly provide a rapid, preliminary evaluation of complex stability and are not sufficient to capture slower conformational rearrangements. Accordingly, we have revised the **Section 3.9** to explicitly state that the short MD simulations for EPHA2, MMP3, and PARP1 provide only initial evidence of binding plausibility and that experimental validation will be necessary to strengthen mechanistic inference.

**(3)** Inclusion of additional stability metrics: 1) We calculated the RMSF to assess protein flexibility. The results (**Figure 10F**) show that residues in the binding pocket exhibit low fluctuation, confirming that ILA binding stabilizes the active site. 2) Protein-ligand interaction analysis was conducted to characterize the persistence and nature of intermolecular contacts throughout the simulation (**Figure 10G**). The analysis revealed that the H-bond with ARG183 was maintained for 81% of the simulation time, providing a structural basis for stable binding. 3) We calculated the binding free energy using the MM/GBSA method. The estimated binding free energy for the ILA-HMOX1 complex was -25.58 kcal/mol. These new data have been integrated into the Results (Section 3.9) and **Figure 10F-G**. Please refer to the revised manuscript for all modifications.

**Reviewer 2, Point 6**

6. Comment: (The statement “All data are available on request from the authors” does not align with PLOS ONE data-sharing recommendations. Public access to analysis scripts (e.g., GitHub, Zenodo) or processed data should be provided where possible. )

**Response:** We sincerely appreciate you for reminding us of the PLOS ONE data-sharing policy. We fully acknowledge that our original statement was insufficient, and we strongly support the journal’s commitment to open science and reproducibility. **(1)** Raw data: The accession numbers and repositories for all public datasets used are explicitly listed. **(2)** Processed data: All processed datasets generated during this study-including the lists of ILA and CRC targets, machine learning importance scores, and pathways and terms for enrichment analysis, have been compiled and uploaded as supplementary information (Supplementary files 1-4). **(3)** We have replaced the "Data Availability Statement" in the manuscript with the following detailed version: All relevant data are within the paper and its supplementary information files.

**Reviewer 2, Point 7**

7. Comment: (Several mechanistic explanations (e.g., AhR/Nrf2 signaling) are repeated across multiple subsections. The Discussion could be streamlined to improve clarity.)

**Response:** We sincerely appreciate you for observing this structural redundancy. To streamline the text and improve flow, we have reorganized and condensed the Discussion section as follows: **(1)** We have consolidated the detailed description of the molecular mechanism by which ILA activates the AhR/Nrf2 pathway into **Section 4.1.1**. This is the most contextually relevant section, given that the hub gene HMOX1 is a direct downstream target of Nrf2. **(2)** We have removed the repetitive introductory text regarding AhR/Nrf2 signaling from **Section 4.2** and **Section 4.3**. These revisions have resulted in a more concise and logically coherent Discussion. Please see the specific modifications in the revised manuscript.

**Reviewer 2, Point 8**

8. Comment: (Minor grammatical errors and overly long sentences are present, particularly in the Discussion. Gene and protein nomenclature should be consistently formatted.)

**Response:** Thank you for your comments. We apologize for not making it clear. In response, we performed a comprehensive language revision of the manuscript, with a particular focus on the Discussion section. **(1)** We corrected grammatical errors and broke down overly long, complex sentences into shorter, more direct statements to enhance readability and logical flow. **(2)** We also standardized gene and protein nomenclature throughout the manuscript to ensure consistency. Abbreviations and full names were also harmonized at first mention and used consistently thereafter. These revisions have been incorporated throughout the manuscript. Please see the specific modifications in the revised manuscript.

We tried our best to improve the manuscript and made some modifications to the manuscript. These minor modifications will not influence the content and framework of the manuscript. Here we did not list these modifications but marked them in red in the revised manuscript.

We appreciate the Editors’ and Reviewers’ hard work earnestly and hope that these modifications and explanations have answered your questions.

Once again, thank you very much for your comments and suggestions.
